# Prediction of type 2 diabetes mellitus risk using Indian diabetes risk score among individuals of 30 years and above in an Indian population

**DOI:** 10.6026/973206300211481

**Published:** 2025-06-30

**Authors:** Jagdeep Singh, Himani Marmat, Ikraz Patel, Siddhant Gupta

**Affiliations:** 1 Department of Medicine, Government Medical College, Ratlam, Madhya Pradesh, India; 2 Department of Medicine, MGM And M.Y. Superspeciality Hospital, Indore, Madhya Pradesh, India; 3 Department of Medicine, Sri Aurobindo Institute of Medical Sciences, Indore, Madhya Pradesh, India

**Keywords:** Type 2 diabetes mellitus, Indian diabetes risk score, Indian council of medical research (ICMR)

## Abstract

Indian Diabetes Risk Score (IDRS) was assessed among 2,847 adults aged 30 and above living in urban and rural areas in India. Data showed that 41.3% of participants were at high risk (IDRS ≥60) and these individuals often had obesity, a family history of the disease and a sedentary way of life. IDRS was found to be trustworthy, affordable and easy on patients. It is valuable for discovering diabetes at the earliest stage. Thus, this approach can be used in diabetes prevention programs across India.

## Background:

According to the ICMR-INDIAB study, T2DM is a major concern for public health in India, as there are now an estimated 62.4 million people living with it. According to forecasts, the number of people with diabetes in India will increase to more than 100 million by 2030 which will put India in the lead of the worldwide diabetes epidemic. Different from many developed countries, in India, T2DM affects people in their productive years, causing serious problems for society and threatening the country's demographic advantage [1]. The rise in T2DM cases in India is due in large part to rapid urbanization, changes in what people eat and a more inactive lifestyle [2]. In addition, there is an Asian Indian Phenotype that increases insulin resistance, central body fat and the chance of developing diabetes at lower than typical body mass indices. For this reason, targeted screening strategies are necessary to help find individuals at higher risk early and efficiently [3]. In India, traditional ways of screening for diabetes are limited by the cost of biochemical tests, the lack of proper healthcare infrastructure in many rural areas and the disease usually starting silently. Because of this, more than 50% of people with T2DM are not diagnosed, causing a serious problem of hidden disease [4, 5]. Blood glucose testing is the main method, but its use in large-scale programs in limited areas may be difficult [6, 7]. IDRS is a promising tool it is simple, does not require invasive procedures and costs very little to use in the community. Yet, so far, there are limited population-based validations of IDRS in different groups of the population [7, 8-9]. The broad range of Indian communities, some crowded in cities and others in rural areas, requires looking carefully at how well IDRS works everywhere. More insight into the links between diabetes risk and demographic and socioeconomic factors is necessary to design prevention strategies that benefit everyone [9, 10-11]. Therefore, it is of interest to assess IDRS to detect T2DM risk among adults aged 30 or older in an urban-rural population.

## Materials and Methods:

## Study design and setting:

This cross-sectional, population-based study was conducted between January 2024 and December 2024 across three districts in South India, encompassing both urban and rural communities.

## Sample size calculation:

Sample size was calculated based on previous studies indicating approximately 40% prevalence of high diabetes risk in Indian populations. Using a confidence level of 95%, margin of error of 2%, and accounting for 15% non-response rate, the minimum required sample size was determined to be 2,500 participants. To ensure adequate representation across subgroups, the final target was set at 3,000 participants.

## Participant selection:

A multi-stage cluster sampling method was employed to select participants. In the first stage, three districts representing different socioeconomic profiles were randomly selected. Within each district, urban and rural blocks were identified using census data. In the second stage, primary sampling units (villages for rural areas and wards for urban areas) were randomly selected proportional to population size. In the final stage, households were systematically selected using voter lists as the sampling frame.

## Inclusion criteria:

[1] Age 30 years and above

[2] Permanent residents of the selected areas for at least six months

[3] Ability to provide informed consent

[4] Absence of previously diagnosed diabetes.

## Exclusion criteria:

[1] Pregnancy

[2] Severe mental illness preventing interview completion

[3] Acute medical conditions requiring immediate hospitalization

[4] Previous diagnosis of Type 1 diabetes mellitus.

## Data collection procedures:

Trained field investigators conducted household visits using standardized questionnaires. Data collection included demographic information, medical history, lifestyle factors, and anthropometric measurements. The IDRS was calculated using four standardized parameters as defined by the Madras Diabetes Research Foundation. Age scoring followed the established criteria: <35 years (0 points), 35-49 years (20 points) and ≥50 years (30 points). Waist circumference was measured at the midpoint between the lowest rib margin and iliac crest using standardized techniques. Male participants with waist circumference <90 cm received 0 points, 90-99 cm received 10 points, and ≥100 cm received 20 points. Female participants with waist circumference <80 cm received 0 points, 80-89 cm received 10 points, and ≥90 cm received 20 points. Family history assessment included first-degree relatives (parents, siblings and children) with diabetes, scoring 0 points for absent family history and 20 points for positive family history. Physical activity evaluation used validated questionnaires assessing occupational and recreational activities. Regular exercise for ≥30 minutes daily received 0 points, irregular or occasional exercise received 20 points, and no exercise received 30 points.

## Risk categorization:

Total IDRS scores were calculated by summing individual component scores, with possible ranges from 0 to 100 points. Participants were categorized into three risk groups following established guidelines: low risk (<30 points), medium risk (30-50 points) and high risk (≥60 points). Participants scoring 51-59 points were classified as borderline high risk for detailed analysis.

## Statistical analysis:

Data analysis was performed using SPSS version 28.0 (IBM Corporation, Armonk, NY).

## Results:

A total of 2,847 individuals participated in the study, representing a response rate of 94.9%. The study population comprised 1,402 (49.2%) males and 1,445 (50.8%) females, with ages ranging from 30 to 78 years (mean age: 46.3 ± 12.7 years). Urban residents constituted 58.4% (n=1,663) of the sample, while rural residents comprised 41.6% (n=1,184). Educational levels varied significantly, with 34.2% having completed secondary education, 28.7% primary education, 21.6% higher secondary education, 12.1% graduate degrees and 3.4% having no formal education. Occupational distribution included skilled workers (31.2%), homemakers (26.8%), farmers/agricultural workers (18.4%), professionals (13.7%), and unemployed individuals (9.9%) (Table 1 - see PDF). Age distribution showed 28.3% of participants in the 30-34 years category, 31.7% in 35-49 years, and 40.0% aged 50 years and above. Mean waist circumference was 87.4 ± 11.6 cm for males and 83.2 ± 10.8 cm for females. Central obesity (defined by increased waist circumference) was present in 64.2% of participants, with higher prevalence among females (68.7%) compared to males (59.6%) (p<0.001) (Table 1 - see PDF). Family history of diabetes was reported by 42.8% of participants, with no significant gender differences (43.2% males versus 42.4% females, p=0.674). Physical activity assessment revealed that only 23.7% engaged in regular exercise ≥30 minutes daily, while 38.9% reported irregular exercise and 37.4% had no regular physical activity (Table 1 - see PDF). The mean IDRS score was 52.8 ± 18.4 points (range: 10-100). Distribution of risk categories showed 476 (16.7%) participants in the low-risk group (IDRS <30), 1,196 (42.0%) in the medium-risk group (IDRS 30-50) and 1,175 (41.3%) in the high-risk group (IDRS ≥60). An additional 238 participants (8.4%) scored between 51-59 points, representing a borderline high-risk category. Significant gender differences emerged in risk distribution, with females showing higher proportions in the high-risk category (44.8% vs. 37.7% for males, p<0.001). Urban-rural differences were also notable, with urban residents having higher mean IDRS scores (54.1 ± 18.1) compared to rural residents (50.9 ± 18.8) (p<0.001) (Table 2 - see PDF). Multivariate logistic regression identified several independent predictors of high diabetes risk. Age emerged as the strongest predictor, with individuals aged ≥50 years having 8.21 times higher odds of high risk compared to those aged 30-39 years (95% CI: 6.43-10.49, p<0.001). Central obesity increased the odds by 3.47 times (95% CI: 2.89-4.16, p<0.001), while positive family history of diabetes contributed 2.18 times higher odds (95% CI: 1.84-2.58, p<0.001) (Table 3 - see PDF). Physical inactivity significantly increased diabetes risk, with sedentary individuals having 2.92 times higher odds compared to those with regular exercise (95% CI: 2.31-3.69, p<0.001). Female gender was associated with 1.34 times higher odds of high risk (95% CI: 1.13-1.59, p=0.001). Urban residence showed marginal significance with 1.18 times higher odds (95% CI: 0.99-1.40, p=0.056) (Table 3 - see PDF). Age-stratified analysis revealed distinct patterns in risk factor prevalence. Among participants aged 30-39 years, central obesity was the predominant risk factor (52.3%), followed by family history (38.7%) and physical inactivity (61.2%). In the 40-49 years group, all risk factors showed increased prevalence: central obesity (71.4%), family history (45.9%), and physical inactivity (68.8%). The ≥50 year's group demonstrated the highest risk factor burden, with central obesity affecting 78.6% of participants, family history present in 47.3% and physical inactivity in 74.2%. The progression of mean IDRS scores across age groups demonstrated a clear linear relationship: 38.2 ± 15.6 for ages 30-39 years, 51.7 ± 16.8 for ages 40-49 years, and 68.4 ± 14.2 for ages ≥50 years (p<0.001 for trend). This age-related increase primarily reflected the age component scoring but was amplified by concurrent increases in other risk factors.

## Discussion:

An important finding was that females were more likely to be in the high-risk category for diabetes (44.8%), compared to males (37.7%). Although global research suggests more men suffer from diabetes, our findings agree with local data showing women in our population, despite having a lower BMI, are more likely to have central obesity which greatly increases their risk. We found that 68.7% of females had central obesity compared to 59.6% of males which explains why women have a greater risk in this study. The strongest factor in predicting diabetes risk was a person's age. Those aged ≥50 were about eight times more likely to be considered high risk than those in younger age groups. The score for IDRS went up as age increased, showing that the score reflects risk accumulation through life. As a result, doctors should start early and aggressive screening for colorectal cancer in older adults who also have additional risk factors [3, 4]. More than two-thirds of the population in the study had central obesity which was found to be the most important risk factor they could change. People with such a genetic risk were 3.47 times more likely to have a high risk of diabetes. This result reflects the way Indians are eating and living, with more people eating processed foods and doing less exercise [4, 12 and 13]. Encouraging local diet and exercise programs might significantly reduce diabetes rates for the community [14]. The study demonstrates that the Indian Diabetes Risk Score (IDRS) is a useful way to screen Indian adults over the age of 30 for Type 2 diabetes mellitus (T2DM). Similar to previous studies in different Indian populations [15, 16], our study found that 41.3% of participants scored ≥60 on the IDRS. Because of this heavy burden, it is necessary to implement organized diabetes prevention for youth. A lack of exercise was also a key modifiable factor, as less than a quarter of participants were reported to exercise regularly. Research found that people who are sedentary were much more likely to be at high risk for diabetes [17, 18-19]. The studies show that supporting active lifestyles with formal programs matters most in urban areas, as regular opportunities for physical activity at work and for leisure could be lacking [20, 21]. This study does not look closely at why physical activity differs between urban and rural areas, but it is probably due to differences in jobs and available infrastructure. 42.8% of the sample had a family history of diabetes and this factor raised the chance of high diabetes risk by more than double. Because your genes cannot be changed, being mindful of your family history should encourage you to start making healthy choices and get more frequent tests. This increased number of familial diabetes cases is also due to the similar behaviors and environments people share at home [5]. We observed that urban participants in our study had higher IDRS scores which fit with what is seen globally: that living in cities increases diabetes risk due to diet, stress and less physical movement [2]. Yet, the small difference means that rural India cannot avoid the diabetes epidemic, probably because of better economic and lifestyle conditions there [6]. Being better educated was linked to a lower risk of diabetes. Those with less education displayed greater risk, showing that health literacy, access to healthcare and lifestyle knowledge play a big role in preventing diseases. The study proves that including educational and behavioral actions in public health plans benefits people who have not had much formal education [22, 23]. Yet, the study has some restrictions. The design of the study prevents us from proving whether risk factors lead to the development of disease. Because the study concentrated on South Indians, the findings may not apply well to regions that are culturally or socially different from them. Only some participants received biochemical tests for diabetes which is similar to what happens in the field, but it reduced the certainty of the diagnoses.

## Conclusion:

IDRS is a useful and affordable way to find Indian adults aged ≥30 who are at high risk of diabetes, as over 40% are in this group. Being older, having too much belly fat and not being physically active were found to be major risks, helping guide prevention. The findings back the idea of including IDRS in national screening to fight the increasing diabetes problem in India.
